# Cerebrovascular flow‐mediated dilation in humans: Methodological challenges, physiological interpretation and future integrations

**DOI:** 10.1113/EP093070

**Published:** 2026-06-22

**Authors:** Yi Zhen Bao, Tabitha V. Craig, Jenna C. McCrone, Kurt J. Smith, Michael M. Tymko

**Affiliations:** ^1^ Department of Human Health Sciences, Integrative Cerebrovascular and Environmental Physiology SB Laboratory University of Guelph Guelph Ontario Canada; ^2^ School of Exercise Science, Physical and Health Education, Cerebrovascular Hemodynamics, Exercise, and Environmental Research Sciences (CHEERS) Laboratory University of Victoria Victoria British Columbia Canada

**Keywords:** flow‐mediated dilation, hypercapnia, internal carotid artery, nitric oxide

## Abstract

Arterial shear‐mediated vasodilation is a well‐established measure of endothelial function and serves as a critical biomarker for cardiovascular disease risk. Endothelial function can be measured using a variety of experimental methodologies; however, the most widely adopted technique is ultrasound‐based flow‐mediated dilation (FMD), in which arterial diameter (e.g., brachial or femoral artery) is quantified before and after a standardized period of blood occlusion using a pressure cuff. Recently, there have been several published studies that have assessed cerebrovascular flow‐mediated dilation (cFMD) in the internal carotid artery (ICA) using transient and steady‐state hypercapnia. Hypercapnia increases cerebral blood flow (CBF), and the consequent increase in arterial shear stress provokes ICA vasodilation. The ICA diameter response is mediated by endothelial derived nitric oxide, like the peripheral vasculature. Since the introduction of the cFMD technique, there have been several early studies investigating the effects of exercise, environment, autonomic nervous activity, age, sex and vasoactive hormones on cerebral endothelial function. While some findings parallel existing literature on peripheral endothelial function or studies primarily measuring shear stress (e.g. effects of ageing), others reveal unique aspects of cFMD regulation that diverge from trends seen in other techniques (e.g., smoking, sitting and handgrip exercise). This review aims to synthesize early findings on the mechanism(s) underlying cFMD, compare methodological approaches, and outline future research directions and potential clinical applications in this emerging field.

## Abstract (highlights)

- **What is the topic of this review?**
  This review summarizes the investigations completed to date on cerebrovascular flow‐mediated dilation in humans as a non‐invasive measure of cerebral endothelial function and highlights potential future research directions and clinical relevance.
- **What advances does it highlight?**
  This review highlights the known physiological modulators of cerebrovascular flow‐mediated dilation and compares findings across methodological approaches, as well as between the cerebral vasculature and peripheral vasculature.

## 1 INTRODUCTION

Cerebral blood flow (CBF) is tightly regulated due to the brain's high metabolic demand and negligible capacity to store fuel. As such, several intrinsic mechanisms independently and synergistically regulate CBF to maintain delivery of oxygen and nutrients, such as myogenic tone (Osol et al., 2002), autonomic nerve activity (Koep et al., 2022), arterial blood gases (oxygen and carbon dioxide content; Willie et al., 2014), intracranial pressure (Wittenberg et al., 2025) and endothelial function (Carter et al., 2016; Hoiland et al., 2017, 2022). The endothelium, which forms the innermost layer of the vasculature, plays a critical role in vasomotor function. Both steady‐state and transient hypercapnia have been employed to increase shear stress on the cerebral endothelium, and the resulting vasodilation within the internal carotid artery (ICA) is thought to be an index of cerebrovascular endothelial function. Indeed, Hoiland et al. (2022) demonstrated that blocking nitric oxide (NO) bioavailability via *N* ^G^‐monomethyl‐l‐arginine (i.e., l‐NMMA) reduces the ICA vasomotor response to a transient increase in shear stress, confirming that this test reflects NO‐mediated cerebral endothelial function. The purpose of this review seeks to consolidate our current knowledge on cerebrovascular flow‐mediated dilation (cFMD), outlining the historical methodologies used to interrogate cFMD in humans, summarizing evidence quantifying its magnitude, and examining how it is influenced by acute interventions, ageing, sex differences and health status.

### 1.1 Brief historical perspective on cerebral blood flow control and cerebral endothelial function

It has been nearly 80 years since Seymour Kety and Carl Schmidt gave us the first look into global cerebrovascular reactivity (CVR) to CO_2_ in humans (Kety & Schmidt, 1948). Since these seminal papers investigating CVR in humans using the Kety–Schmidt technique, CBF has been largely defined by its sensitivity to CO_2_, with hypercapnic increases in CBF attributed to reductions in microvascular resistance and increased capillary recruitment (reviewed in Willie et al., 2014). However, one of the primary disadvantages with the Kety–Schmidt technique was its low temporal resolution making it difficult to explore the mechanisms responsible for hypercapnic induced hyperaemia. Advancements in cerebrovascular imaging using ultrasound in the 1980s (Aaslid et al., 1982), as well as in vitro experimental techniques demonstrating the necessity of endothelial cells in cholinergic vasodilation (Furchgott & Zawadzki, 1980), provided improved spatial and temporal resolutions in measuring dynamic and complex physiological mechanisms and highlighted the critical role of endothelial function in cerebrovascular regulation.

Foundational insights into endothelial responses to chemical and haemodynamic stimuli helped establish the physiological basis from which cFMD would later emerge. Furchgott & Zawadski (1980) demonstrated that the descending thoracic aorta only vasodilates in response to acetylcholine when the endothelium was intact but vasoconstricted when absent. Shortly after, it was revealed by others, that haemodynamics (i.e., changes in blood flow) that mechanically stimulate the endothelium upregulated paracrine hormones, producing molecules from the endothelium that caused a vasodilatory response (Pohl et al., 1986; Rubanyi et al., 1986). These findings ultimately led to the seminal work by Celermajer et al. (1992) that highlighted the relationship between peripheral endothelial shear‐mediated vasodilation and NO, as well as the cardiovascular risk factors associated with endothelial dysfunction using dynamic ultrasound imaging (Celermajer et al., 1992). At the time, despite vascular duplex ultrasound (VDu) being available, no studies using these techniques were used to measure contemporaneous diameter and velocity profiles to assess flow and shear rates through extracranial cerebral arteries.

However, using transcranial Doppler (TCD) ultrasound (Aaslid et al., 1982), rapid and robust beat‐by‐beat changes in cerebral blood velocity (a surrogate for CBF) in response to manipulations in arterial blood gases were regularly performed. Using TCD to assess regional intracranial CVR in vessels projecting from the circle of Willis (middle and posterior cerebral arteries) (Skow et al., 2013), these studies typically integrate TCD and arterial blood pressure responses during hypercapnia to to infer changes in cerebrovascular reactivity and downstream vascular resistance/conductance. (Willie et al., 2012).

A limitation of TCD, despite its beneficial temporal resolution, is that it was unable to quantify vessel cross‐sectional area and, thus, cannot quantify the haemodynamic stimuli responsible for shear‐mediated vasodilation (i.e., diameter, flow and shear). Nevertheless, using TCD, researchers have utilized the physiological responses during CVR tests to observe that cerebrovascular haemodynamics occur within 10–20 s upon inspiration of elevated CO_2_. Fortunately, the high temporal and spatial resolutions provided by VDu when paired with advanced analysis software make it possible to assess beat‐by‐beat changes in flow, shear and blood vessel diameter forming the basis for the quantification of endothelial function within the cerebral vasculature. Collectively, utilizing TCD and VDu imaging to delineate the intra‐ and extra‐cranial physiological responses during CVR tests (Sato et al., 2012; Willie et al., 2012), researchers have been able to isolate shear‐mediated haemodynamics in extracranial arteries (reviewed in Tables 1 and 2).

**TABLE 1:** Summary of studies conducted in humans examining how physiological factors influence transient cFMD

Study	Population	Intervention	Sample	CO_2_ controller system; CO_2_ severity; and CO_2_ duration	ICA diameter change (%); ICA normalization	Shear rate correlation with ICA dilation	Summary
Saito et al. (2023)	Young and healthy adults	Isometric handgrip exercise	13 (10M/3F)	Gas blender 20 breaths/min paced with metronome +9 mmHg 30 s	NS Normalized for SR_AUC_	NS	Isometric handgrip exercise had no effect on transient cFMD
Sakamoto (2024)	Young and healthy adults	Acute aerobic exercise	16 (9M/7F)	Gas blender 20 breaths/min +10 mmHg 30 s	Baseline: 3.32 ± 1.37 Spontaneous breathing: 4.74 ± 1.84 (*P* < 0.01) Baseline: 3.35 ± 1.15 Hyperventilation and CO_2_: 4.33 ± 2.12 (*P* = 0.04) Normalized for *D*_base_ and SR_AUC_	Positive correlation	Acute aerobic exercise increases transient cFMD 10 min post‐exercise, compared to control and baseline
Saito et al. (2025)	Young and healthy adults	Prolonged sitting	13 (10M/3F)	Gas blender 20 breaths/min paced with metronome +9 mmHg 30 s	NS Normalized for SR_AUC_	NS	Prolonged sitting showed no effect on transient cFMD
Walsh et al. (2025)	Young and healthy adults	Interval vs. continuous exercise	14 (M)	Gas blender 20 breaths/min paced with metronome +7 mmHg 30 s	Ns – for either exercise Normalized for *D*_base_ and SR_AUC_	NA	Neither exercise type elicited significant change in transient cFMD

Abbreviations: *D*_base_, baseline diameter; NA, not applicable; NS, no significant difference; SR_AUC_, shear rate area under the curve.

**TABLE 2:** Summary of studies conducted in humans examining how physiological factors influence steady‐state cFMD.

Study	Population	Intervention	Sample	CO_2_ controller system; CO_2_ severity; and CO_2_ duration	ICA diameter change (%); cFMD normalization	Shear rate correlation with ICA dilation	Summary
Iwamoto et al. (2018a)	Young and healthy adults	Acute SNS (+20 mmHg LBNP)	9 (M)	Partial‐rebreathe and gas blender +10 mmHg 3 min	Control: 5.5 ± 0.7 LBNP: 1.8 ± 0.4 (*P* < 0.01) Normalized for *D*_base_ and SR_AUC_	NS	Steady state cFMD is attenuated during the final 3 min of acute SNS activation compared to control
Iwamoto et al. (2018b)	Young and older adults	Rest	10 Young (5M/5F) 10 Old (4M/6F)	Partial‐rebreathe and gas blender +10 mmHg 3 min	Young: 7.9 ± 1.2 Older: 4.5 ± 0.5 (*P* < 0.01) Normalized for *D*_base_ and SR_AUC_	Positive correlation	Older adults exhibited lower steady state cFMD compared to young adults at +10 mmHg CO_2_
Suzuki et al. (2020)	Young habitual cigarette smokers	Rest	10 non‐smokers (10M), 10 smokers (9M/1F)	Gas blender +10 mmHg 3 min	NS No *D*_base_ or SR_AUC_ normalization	NS	Habitual cigarette smoking does not affect steady state cFMD
Iwamoto et al. (2020)	Young and healthy adults	Intermittent hypoxia	9 (8M/1F)	Partial‐rebreathe and gas blender +10 mmHg 3 min	Baseline: 4.6 ± 1.3 Post‐IH: 6.2 ± 2.2 (*P* < 0.01) Normalized for SR_AUC_	Positive correlation	Intermittent hypoxic training increases steady state cFMD 20 min post‐stimulation
Iwamoto et al. (2021)	Healthy women 2 menstrual phases Pre‐ versus peri‐ versus post‐menopause	Rest	11 pre‐menopause, 13 peri‐menopause, 10 post‐menopause	Nonrebreathing system +10 mmHg 3 min	EF: 6.4 ± 1.1% LF: 8.9 ± 1.4%, Peri‐menopause: 5.5 ± 1.3% Post‐menopause: 5.2 ± 1.9% All *P* < 0.05 except peri‐menopause versus post‐M Normalized for *D*_base_ and SR_AUC_	NS	Participants with lower estrogen levels during EF, Peri‐menopause, and post‐menopause showed reduced steady state cFMD Lower serum oestradiol concentration also correlates with reduced steady state cFMD
Sakamoto et al. (2021)	Young and healthy adults	High (HIE) and moderate (MIE) acute aerobic exercise	12 (M)	Gas blender +10 mmHg 3 min	Baseline: 6.9 ± 1.7% HIE: 4.0 ± 1.4% (*P* < 0.01) MIE: NS Normalized for *D*_base_ and SR_AUC_	NS	High intensity exercise acutely decreases steady state cFMD 5 min post‐exercise Moderate intensity exercise showed no changes in steady state cFMD
Tallon et al. (2023)	Children	Rest	14 children (6M/8F) 17 adults (10M/7F)	Douglas bag 6% CO_2_ 4 min	NS No *D*_base_ or SR_AUC_ normalization	NS	Children showed no difference in steady state cFMD, but slower mean response time in SR, blood flow and velocity
Sakamoto (2022)	Young and healthy adults	Dynamic resistance exercise	15 (11M/4F)	Gas blender +10 mmHg 3 min	NS Normalized for *D*_base_ and SR_AUC_	NS	Dynamic resistance exercise showed no changes in steady state cFMD
Freeberg et al. (2025)	Young adults and older adults	Vitamin C i.v. infusion	20 young (10M/10F), 21 older (10M/11F)	Douglas bag 5% CO_2_ 4 min	Young: NS Older adults: 68 ± 1 years Saline: 4.3 ± 2.4% Vitamin C: 6.7 ± 3.3% Normalized for *D*_base_, SR_peak,_ and SR_AUC_	NS	Older adults showed increased steady state cFMD during vitamin C infusion, while young adults did not

Abbreviations: *D*_base_, baseline diameter; EF, early follicular phase; F, female; LF, late follicular phase; M, male; NS, no significant difference; SRAUC, shear rate area under the curve; SRpeak, peak shear rate.

**FIGURE 1:**
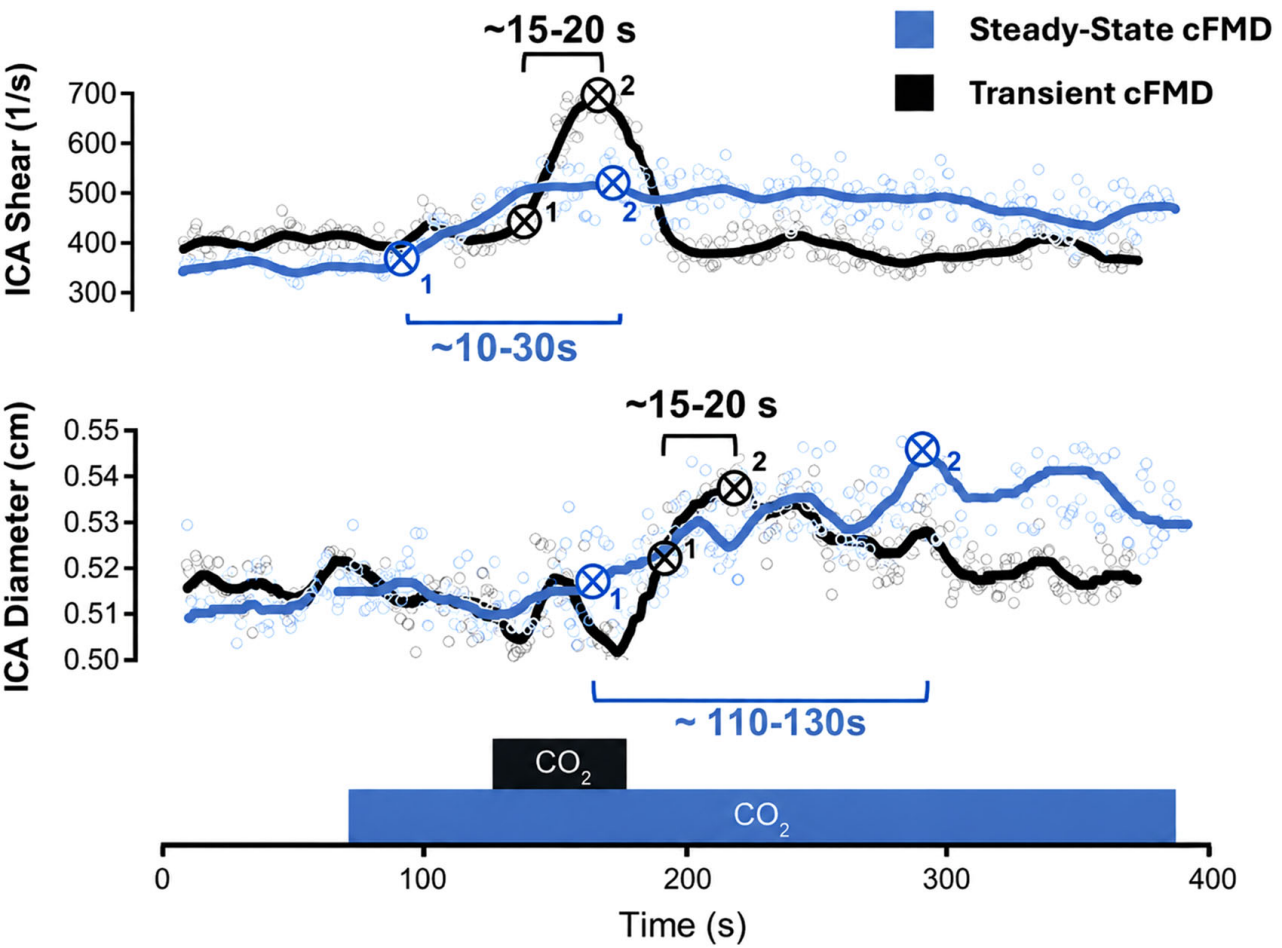
Illustration of steady‐state (blue) and transient (black) CO_2_ cerebrovascular flow‐mediated dilation test. Steady‐state, similar to the approach used by Carter et al., 2016, and transient ICA shear rate (upper panel) and ICA diameter (bottom panel) responses observed by Hoiland et al. (2017).

The first study to showcase cerebral endothelial function (Carter et al., 2016) built upon an earlier study that identified the potential of intra‐ and extra‐cranial cerebral artery vasodilation during iso‐oxic hypercapnia by comparing relative changes in blood velocity and vessel diameter between the ICA and middle cerebral artery (MCA) (Willie et al., 2012). Carter et al. (2016) demonstrated the effectiveness of using CO_2_ (e.g., steady‐state hypercapnia for 4‐min) to assess increased shear‐mediated vasodilation in the ICA and common carotid artery (CCA), providing mechanistic observations of dilation in the cerebral arteries previously hypothesized by Willie et al. (2012), and evidenced by Coverdale et al. (2014) and Verbree et al. (2014). Notably, Carter et al. (2016) used the steady state hypercapnic test to demonstrate a significant positive correlation (*r* = 0.679, *P* < 0.01) between % Δ in ICA diameter and % Δ in shear rate. Further, the ICA dilation (81 ± 51 s), temporally follows hyperaemic increases in MCA blood velocity (MCAv) (18 ± 9 s) and ICA shear rate (22 ± 12 s). This is suggestive of both structural and functional characteristics of the cerebral hypercapnic haemodynamic responses. Firstly, because the MCAv hyperaemia occurs prior to ICA shear, it infers that distal conductive vasodilation initiates the shear‐mediated intra‐ and extracranial haemodynamics. Secondly, the latency of the ICA dilation in relation to the preceding ICA hyperaemia is similar to the latencies observed during brachial artery FMD (Black et al., 2008). Collectively, this study was the first to provide in vivo evidence that hypercapnic cerebrovascular haemodynamics can be used to assess cerebral endothelial function. The following year, Hoiland et al. (2017) used a 30‐s CO_2_ test (+9 mmHg CO_2_) and compared the responses to the steady state CO_2_ test utilized by Carter et al. (2016). The hypothesized benefit of transient hypercapnia mitigated the potential confounding influences of hypercapnic‐induced hypertension observed during the steady state CO_2_ test. This transient CO_2_ test resulted in ICA vasodilation immediately following the CO_2_‐induced increases in shear rate, and they occurred earlier compared to the steady state CO_2_ vasodilation test (see Figure 1).

Furthermore, the transient CO_2_ test (i.e., cFMD) exhibited a significant correlation between shear rate area under the curve (SR_AUC_) to percentage ICA vasodilation. This relationship was not observed in response to a steady state CO_2_ test. The SR_AUC_ has since been observed as being an important factor in establishing reliable cFMD responses (Sakamoto et al., 2025). The transient CO_2_ test also elicits faster (59.4 ± 60.3 vs.110.3 ± 79.6 s, *P* = 0.047), yet lower (−60%, *P* = 0.03) peak shear rate and percentage dilation responses. In a follow up study, Hoiland et al. (2022) demonstrated that the transient CO_2_ vasodilatory response is NO‐mediated, since NO inhibitor (l‐NMMA) reduced ICA dilation by 37%. This occurred despite greater increases in shear rate during the l‐NMMA condition compared to placebo condition. It is worth mentioning that some of the vasodilation during the steady state CO_2_ test may also be NO‐mediated; however, this has not been experimentally quantified. Since these important studies have been completed, several research teams have published data showcasing the importance of cerebrovascular endothelial function using both transient and steady state CO_2_ tests. Despite, the concerns of steady state tests, the studies discussed in this review will include both transient and steady state CO_2_ tests as measures of cFMD.

While CVR and cFMD inform about cerebrovascular haemodynamics in response to CO_2_, ultimately, they provide different regulatory information. For instance, cFMD informs about the function (i.e., shear‐mediated vasodilation) of the ICA using VDu. However, CVR informs about the whole brain, macrovascular and microvascular, haemodynamic regulation of CBF, and has in the past relied more heavily on TCD velocity measures. While these tests appear similar to one another, the cFMD test only includes a transient single stage of hypercapnia, while CVR tests often include multiple stages in hypo‐ and hypercapnic ranges, and concomitantly increases heart rate and blood pressure. Both cerebrovascular metrics, depending on the imaging used, inform about brain blood flow (e.g., VDu) and haemodynamics (e.g., TCD) regulation; however, only the transient hypercapnic cFMD test has been confirmed to be endothelial nitric oxide mediated (Hoiland et al., 2022). Given that cFMD is fundamentally interpreted as a shear‐mediated endothelial response, the following section outlines the physiological basis by which shear stress is transduced into vasodilation.

### 1.2 Shear‐mediated endothelial function

**FIGURE 2:**
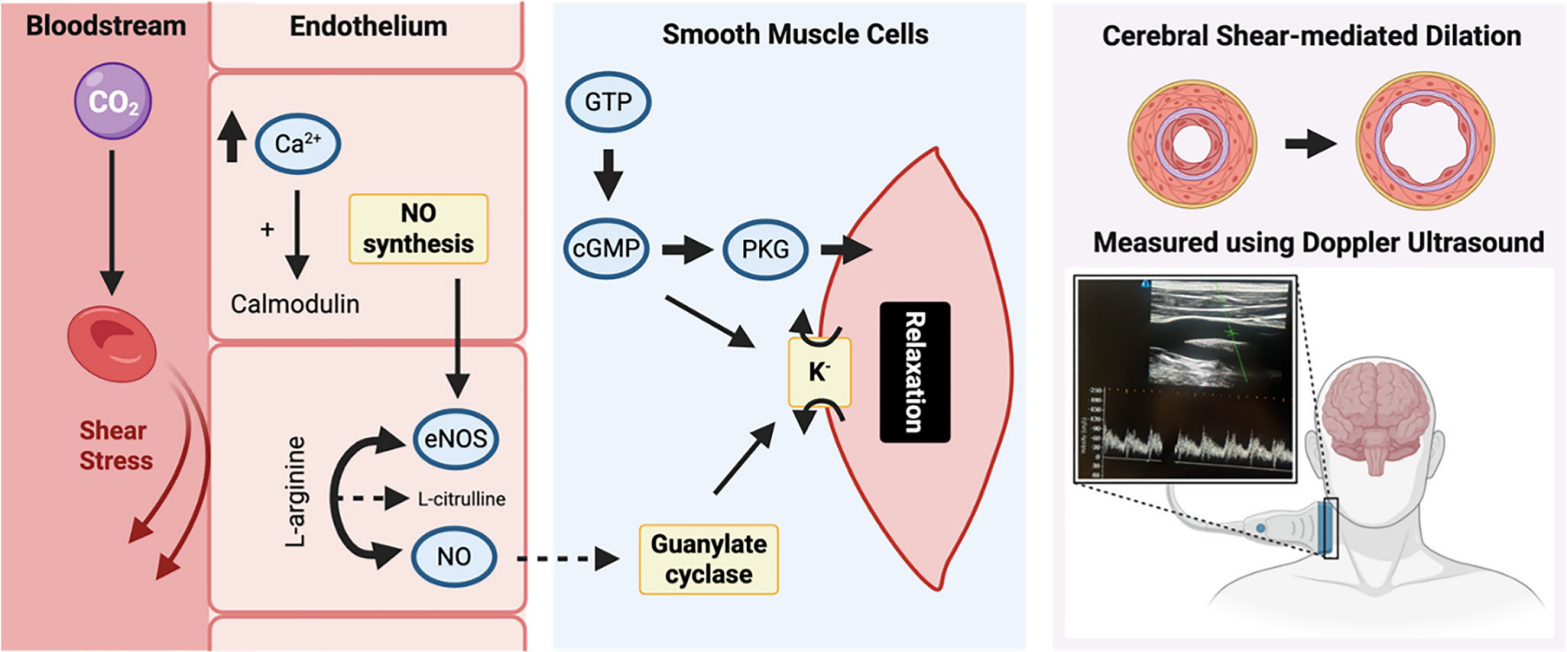
Illustration of shear stress‐mediated NO release resulting in smooth muscle relaxation. Refer to text for an overview of these mechanism(s).

Shear stress on the inner blood vessel wall is a measure of the frictional force applied by red blood cells as they interact with the inner lining of the arteries (i.e., endothelium) (see Figure 2). In the absence of conditions that significantly alter blood viscosity (e.g., high altitude, sickle cell anaemia, dehydration or chronic inflammation), changes in shear rate, estimated as 4 × mean blood velocity/diameter, are reflective of changes in shear stress (Baker & Wayland, 1974). Stimulation of endothelial mechanoreceptors (e.g. GPCRs, PIEZO1 and NOTCH1) and structures (e.g., cilia) produce a biochemical cascade, initiating an influx of Ca^2+^ and enabling endothelial NO synthase (eNOS) to release NO via phosphoinositide 3‐kinase/AKT signalling (Mierke, 2024). On the apical membrane of the endothelium lies a layer saturated with glycoproteins and proteoglycans, termed the glycocalyx, which enhances the coupling of the NO cascade (Cheng et al., 2025). NO is a potent vasodilator that stimulates soluble guanylate cyclase, eliciting increased cGMP synthesis from GTP, sustaining K^+^ efflux, and subsequent smooth muscle relaxation (Guerra et al., 2018).

## 2 FACTORS THAT INFLUENCE CEREBRAL ENDOTHELIAL FUNCTION

This section synthesizes current evidence on physiological modulators of cFMD. Investigations have examined acute interventions, such as sedentary behaviour, exercise, sympathetic activity, intermittent hypoxia, oxidative stress, and long‐term effects such as ageing, sex hormones and smoking, with effects that vary across protocols, populations and measurement approaches. We summarize the principal findings and methodological considerations for each factor. For clarity, we have referred to cFMD studies as transient (∼30 s CO_2_ test) and steady state (sustained CO_2_ test of several minutes). The studies outlined below are summarized in Tables 1 and 2.

### 2.1 Sedentary behaviour: Transient and steady state cFMD tests

With the increasing sedentary behaviours, specifically prolonged sitting with the prevalence of ‘office’ work, daily life inactivity has become independently associated with health risks, including cardiovascular and cerebrovascular disease. A recent study by Saito et al. (2025) explored this; they sought to investigate whether transient cFMD would be altered by prolonged sitting up to 4 h in adults. The authors investigated both transient cFMD (+9 mmHg partial pressure of end‐tidal CO_2_ ($P_{\mathrm{ETC}O_{2}}$)), and peripheral FMD (popliteal artery) at every hour of continuous sitting without movement of the lower limbs. While FMD in the popliteal artery decreased by ∼50% after only ∼2 h, transient cFMD remained unchanged. The disparity between peripheral and cerebrovascular endothelial function during sedentary activity are attributed to the preserved ICA shear stress observed during sitting. The potential for regional differences in endothelial function between the peripheral and cerebral vascular beds likely hinges on the impact of altered shear profiles, especially considering the high shear rates observed in the brain compared to peripheral arteries like the brachial or femoral (Carr et al., 2020; Saito et al., 2025).

No study has officially investigated whether sedentary activity influences steady state cFMD outcomes. Tallon et al. (2023) conducted a study investigating the benefits of exercise breaks during sedentary sitting (i.e., 3 h) on CVR to CO_2_ in children (Tallon et al., 2023). The protocol consisted of breathing a hypercapnic inspirate for 4 min, while ICA shear rate, blood flow and diameter were measured. Similar, to the observations by Saito et al. (2025), Tallon et al. (2023) did not observe any significant changes in ICA shear rate and ICA dilation following sedentary activity. They did observe a reduction in reactive hyperaemic ICA flow (34%) and a delay in the hyperaemic onset (12 s), compared to responses observed when sedentary activity time was interrupted by brief bouts of cycling exercise. A secondary analysis of their steady state hypercapnic ICA dilations normalized for the change in shear rate suggests a lower dilation per unit shear following sedentary activity (6.06 × 10^−^^4^) compared to sedentary time interrupted with physical activity breaks (1.10 × 10^−^^3^). While it should be stated that the Tallon et al. (2023) outcomes do not represent a formal assessment of shear‐mediated dilation, they do raise the possibility that exercise breaks during prolonged sedentary activity may alter temporal dynamics and impact steady‐state cFMD outcomes.

Although higher daily sedentary activity (>452 min) is associated with lower resting ICA shear rate in children and adolescence (Tallon et al., 2021), acute bouts of sedentary activity (e.g., 4 h of sitting) do not show significant changes in steady state cFMD in adults (Saito et al., 2025). It remains to be determined whether other patterns of sedentary behaviour, such as typical office work with intermittent sitting, also alter resting cerebral shear rates and/or if they impact steady‐state or transient cFMD responses differentially.

#### 2.1.1 Exercise: Transient cFMD tests

Exercise is an important regulator of brain health. Specifically, it has been observed to influence cerebrovascular health. An important factor to consider is how exercise intensity and modality influence cerebrovascular shear stress. Similar to the peripheral vessels (Birk et al., 2012; Green et al., 2017; Tinken et al., 2010), to elicit a significant endothelial benefit, it is pertinent that the exercise intervention stimulates the endothelial cells. Recently several studies have completed acute exercise studies and explored their impact on cerebrovascular shear‐mediated vasodilation using transient cFMD tests.

A study by Saito et al. (2023) explored whether handgrip exercise influenced transient cFMD and cognition. The authors used a transient CO_2_ protocol (+9 mmHg CO_2_) combined with cognitive tasks, at 5 min and 60 min post handgrip exercise. They found that transient cFMD did not change despite improved cognitive response times. These findings indicate that the cognitive improvements associated with handgrip exercise are not endothelial‐mediated. An explanation provided by the authors is that the handgrip task may increase sympathetic nerve activity post‐exercise (Saito et al., 2023), which may obscure potential improvements in shear‐mediated cFMD. It is also plausible, given that handgrip exercise did not elicit a cerebrovascular shear stimulus, that it altered cerebral artery endothelial function. Future studies exploring if resistance exercise can elicit changes to endothelial function should ensure that the intervention stimulates endothelial pathways.

In contrast to handgrip exercise, a later study by Sakamoto et al. (2024) reported in 16 participants that 30 min of aerobic cycling at 80% of the ventilatory threshold significantly increased transient cFMD (+10 mmHg $P_{\mathrm{ETC}O_{2}}$) 5 min post‐exercise, indicating that cerebral endothelial function is immediately elevated after acute cycling exercise. A study by Walsh et al. (2025) expanded on these data by comparing transient cFMD responses during moderate‐intensity, workload‐matched continuous and interval semi‐recumbent cycling exercise in 14 healthy participants. The authors observed that transient cFMD (provoked using a slightly lower stimulus of +7 mmHg $P_{\mathrm{ETC}O_{2}}$), did not elicit a significant post‐exercise response despite a 25% improvement in cFMD after 40 min of recovery from continuous exercise, and a 14% improvement following 15 min of recovery from interval exercise (Walsh et al., 2025). While no differences in ICA diameter were noted following exercise in the study by Walsh et al. (2025), lower baseline shear rates were observed. Smith et al. (2019) also reported lower shear rates in the ICA following 30 min of sub‐maximal exercise and hypercapnia. Sustained vasodilations in the ICA following the two hyperaemic conditions were posited to impact post‐exercise shear rates (Smith et al., 2019). Whether lower shear rates influenced cFMD responses remains to be determined and further highlights the importance of normalizing cFMD for shear rate.

Another possible explanation for the disparity between studies relates to differences in the methodology used to stimulate shear rate and CO_2_ across protocols. Sakamoto et al. (2024) directly manipulated cerebral shear during exercise, focusing on a consistent and sustained magnitude and duration of shear rate hyperaemia. Using shear rate as the independent variable experimentally provides a clearer dose–response relationship between shear rate and CO_2_ outcomes. In contrast, Walsh et al. (2025) adjusted workload between exercise conditions, which resulted in differential shear rates being observed between interval (increased shear rate) and continuous (minimal change) exercise. Thus, the experimental design may have impacted the different outcomes observed by these two interesting exercise and cerebrovascular endothelial function studies. Additionally, Sakamoto et al. (2024) applied a transient hypercapnic stimulus of +10 mmHg $P_{\mathrm{ETC}O_{2}}$ above baseline, whereas Walsh et al. (2025) used a smaller increase of ∼7 mmHg $P_{\mathrm{ETC}O_{2}}$. Since elevations in the partial pressure of arterial CO_2_/$P_{\mathrm{ETC}O_{2}}$ are key contributors to increases in CBF and endothelial shear, these methodological differences may have influenced the magnitude of the shear stimulus during cFMD testing. However, the more plausible explanation is that differences in the shear stimulus associated with the exercise protocols contributed to the discrepant findings.

Collectively, these data indicate that exercise modality and intensities that stimulate increases in shear may impact extracranial vasomotor sensitivities during transient cFMD tests. However, continued research is needed to examine how different exercise modalities (e.g., resistance vs. aerobic, and continuous vs. interval) influence cerebral vasculature both acutely and chronically if interventions aiming to optimize exercise to advance cerebrovascular improvements are successful. This deeper understanding is critical for predicting long‐term adaptations and informing evidence‐based exercise recommendations to support brain health.

### 2.2 Exercise: Steady‐state cFMD tests

Sakamoto et al. (2021) investigated the influence of aerobic exercise intensity on steady state cFMD using a protocol of 3 min of hypercapnia at +10 mmHg $P_{\mathrm{ETC}O_{2}}$. Twelve young, healthy males completed 30‐min cycling trials at both high‐intensity exercise (85 ± 5% of max heart rate) and moderate‐intensity exercise (65 ± 5% of max heart rate). Compared to baseline, steady state cFMD was significantly attenuated 5 min post‐exercise following high intensity exercise (6.9 ± 1.7% to 4.0 ± 1.4%, *P* < 0.05), whereas moderate intensity exercise induced no significant changes in steady state cFMD (Sakamoto et al., 2021). Notably, shear rate during exercise did not differ from pre‐exercise, nor at any point between conditions, and steady state cFMD returned to baseline within 60 min post‐exercise. Proposed mechanism(s) for the post‐high intensity exercise attenuation in steady state cFMD including heightened sympathetic nervous system activation and hyperventilation‐induced hypocapnia increased production of reactive oxygen species leading to NO scavenging.

In a follow‐up study, Sakamoto et al. (2023) examined the effects of resistance training on steady state cFMD using a 3‐min +10 mmHg $P_{\mathrm{ETC}O_{2}}$ protocol. Fifteen young, healthy adults completed five sets of leg extensions, and steady state cFMD was measured before and after 10 min of resistance exercise. Regardless of exercise intensity or the number of repetitions per set, neither shear rate between exercise sets nor steady‐state cFMD was altered post‐exercise compared to baseline (Sakamoto et al., 2023). Only one study to date has compared the impact of continued (3 months) resistance or endurance exercise on steady state cFMD (Thomas et al., 2021). In a unique randomized cross‐over training study (3 months of exercise, 1 month washout and a further 3 months of exercise), Thomas et al. (2021) observed reduced ICA vascular resistance during 3 min of steady‐state hypercapnia following resistance, but not endurance, exercise training in 34 monozygotic and dizygotic twin pairs (*n* = 68). However, no differences were observed in shear‐mediated ICA vasodilation following either endurance or resistance exercise training.

Findings from these research studies highlight how acute exercise impacts steady state cFMD, and the importance of exercise modality and intensity on the response. To advance the field, standardized approaches for quantifying cFMD are needed. Nearly 30 years ago, Green et al. (1994) emphasized the importance of exercise‐induced haemodynamic stimuli in peripheral arteries, where repetitive and episodic increases in shear stress drive vascular adaptations (Green et al., 1994). However, it remains unclear whether the same observable training effect can occur in the cerebral vasculature, and whether there are different exercise modalities that are more effective at altering shear within the cerebral vasculature compared to the peripheral vasculature.

#### 2.2.1 Sympathetic nervous activity: Steady‐state cFMD tests

Elevated sympathetic nervous activity (SNA) is a hallmark in numerous disease states such as heart failure, atherosclerosis, ageing, obesity and chronic obstructive pulmonary disease (Fisher et al., 2009). In young and healthy individuals, increasing SNA acutely can be achieved using a number of methodologies, such as hypoxia, exercise and lower body negative pressure (LBNP). In the periphery, elevated SNA attenuates brachial artery FMD (Hijmering et al., 2002). To our knowledge, only one study has attempted to isolate the impact of SNA on steady state cFMD. Iwamoto et al. (2018a) measured steady state cFMD in nine young and healthy males, while being subjected to −20 mmHg of sustained LBNP (5 min). During the LBNP, +10 mmHg $P_{\mathrm{ETC}O_{2}}$ of steady‐state hypercapnia was introduced during the final 3 min. The authors found that steady state ICA cFMD, but not steady state CCA cFMD, was reduced during LBNP, indicating that acutely elevated SNA reduces cerebral endothelial function (Iwamoto et al., 2018a). These findings parallel results in the peripheral circulation, where Tymko et al. (2017) reported that increasing SNA through moderate‐intensity exercise reduced post‐exercise brachial artery endothelial function via an α‐adrenergic pathway (Tymko et al., 2017). Future work should investigate if transient cFMD test response is augmented when experimentally incorporating sympathetic antagonists such as prazosin (Carr et al., 2022). If cFMD improves under SNA inhibition, this would provide experimental evidence of a relationship between SNA and cFMD during LBNP.

#### 2.2.2 Intermittent hypoxia: Steady‐state cFMD tests

In response to hypoxia, blood vessels elicit a compensatory dilatory response to increase blood flow to sustain adequate oxygen delivery to tissue. Iwamoto et al. (2020) focused on steady state cFMD to cycles of intermittent hypoxia. The author subjected nine young adults who described themselves as sedentary to recreationally active to 5 isocapnic intermittent hypoxia cycles with a target $S_{pO_{2}}$ of ∼80%, each lasting 10 min in duration (Iwamoto et al., 2020). ICA blood flow and shear rate both increased during intermittent hypoxia, and steady state cFMD (3 min of hypercapnia at +10 mmHg $P_{\mathrm{ETC}O_{2}}$) was measured at baseline and again 20 min after intermittent hypoxia exposure. The authors found a significant increase in steady state cFMD in the ICA (4.6 ± 1.3% to 6.2 ± 2.2%, *P* < 0.01) and attributed their findings to the overpowering hypoxic vasodilatory response compared to combating the vasoconstrictive effects of hyper‐SNA (see SNA related section above for more detail) during intermittent hypoxia. Intermittent hypoxia therapy is an exciting new area of research that may provide some level of vascular benefits (Casey & Joyner, 2012). Future work should consider characterizing intermittent hypoxia training by determining the optimal hypoxia stimulus (e.g., severity, length and number of cycles) to maximize endothelial benefits.

### 2.3 Oxidative stress: Steady‐state cFMD tests

Conditions that support an imbalance of reactive oxygen species (e.g., superoxide) and antioxidants (Seals et al., 2011) can promote harmful oxidative stress leading to an impairment of cerebrovascular endothelial function due to NO scavenging. Freeberg et al. (2025) infused ascorbic acid to younger (23 ± 3 years) and older (69 ± 9 years) adults to acutely reduce oxidative stress. Using a steady‐state 4‐min 5% CO_2_ protocol before and after infusion, they found that steady state cFMD significantly increased in older adults following vitamin C (saline: 4.3 ± 2.4% vs. vitamin C: 6.7 ± 3.3%, *P* = 0.002), whereas steady state cFMD in younger adults was unchanged (Freeberg et al., 2025). These findings highlight oxidative stress as a key contributor to impaired cerebrovascular endothelial function in ageing, with vitamin C improving steady state cFMD only in older adults, where reactive oxygen species are expected to be greater. Future studies should explore whether chronic or targeted antioxidant strategies can provide sustained cerebrovascular benefits and reduce age‐related vascular risk.

#### 2.3.1 Ageing: Steady‐state cFMD tests

Cardiovascular and cerebrovascular function declines with age for a number of physiological reasons outlined elsewhere (Seals et al., 2014; Zimmerman et al., 2021). Iwamoto et al. (2018b) followed up previous work to explore the relationship between ageing and cerebrovascular endothelial health. Young (*n* = 10; 23 ± 1 years) and older (*n* = 10; 68 ± 1 years) adults were exposed to +5 mmHg and +10 mmHg $P_{\mathrm{ETC}O_{2}}$ for 3 min. In the older group, steady state cFMD was significantly lower during the +10 mmHg stimulus compared to the younger group (4.5 ± 0.5 vs. 7.9 ± 1.2%, *P* < 0.01) (Iwamoto et al., 2018b). The observed decline in steady state cFMD is similar to what has been reported in the peripheral vasculature (Celermajer et al., 1994).

Although adult populations have been widely studied, developmental changes in steady state cFMD remain poorly understood (Thijssen et al., 2009). Tallon et al. (2022) aimed to investigate these relationships by comparing steady state cFMD between 14 children (9.8 ± 0.7 years) and 17 adults (24.7 ± 1.8 years). The authors found that steady state cFMD in the ICA was the same between children and adults; however, children exhibited notably longer ICA response times to the onset of hypercapnia in measures of peak flow (108 s vs. 66 s), velocity (120 s vs. 52 s) and shear rate (90 s vs. 47 s) (Tallon et al., 2022). The longer time to peak blood flow, velocity, and shear responses in children may be a product of the higher baseline blood flows observed in children compared to adults, which could influence steady‐state cFMD. Indeed, CBF has been shown to increase from infancy through late childhood, and then decline beginning in early adolescence, around 12 years of age (Paniukov et al., 2020; Satterthwaite et al., 2014). Further research is necessary to clarify how endothelial responsiveness develops, and future cFMD studies should account for age‐related physiological differences in paediatric populations.

#### 2.3.2 Sex: Steady‐state cFMD tests

Oestrogen is a crucial vasodilator and protective hormone against vascular diseases and associated risk factors (Mendelsohn & Karas, 1999). Iwamoto et al. (2021) examined how fluctuations in oestradiol levels during the menstrual cycle and across menopause stages (peri‐ and post‐menopause) influence steady state cFMD. The authors found that early follicular phases, corresponding with low oestradiol, had a significantly lower steady state cFMD (6.4 ± 1.1%) compared to the late follicular phase, which typically has higher oestradiol (8.9 ± 1.4%, *P* < 0.05). Subsequently, peri‐menopause (5.5 ± 1.3%, *P* < 0.05) and post‐menopause (5.2 ± 1.9%, *P* < 0.05) females demonstrated significantly attenuated steady state cFMD compared to all premenopausal females (Iwamoto et al., 2021). Moreover, SR_AUC_ did not differ between participant groups, and only post‐menopausal females had significantly lower baseline shear rate. Serum oestradiol levels were also measured in this study, which showed a positive correlation with steady state cFMD (*r* = 0.55, *P* < 0.01 and *r* = 0.35, *P* < 0.02 after adjusting for age). Overall, these findings demonstrate the influential role of oestrogen in cerebrovascular function and reinforce the importance of controlling for menstrual phases in future studies involving female participants.

#### 2.3.3 Smoking: Steady‐state cFMD

Habitual cigarette smoking is a profound risk factor for a plethora of diseases, partly through impairment of the systemic vasculature and the induction of hypertension (Gallucci et al., 2020; Wald & Hackshaw, 1996). A clear association exists between active smoking and elevated risks of cerebral ischaemia and stroke (Shah & Cole, 2010). Impaired vascular adaptations associated with smoking are thought to be the result of reduced NO bioavailability and increased reactive oxygen species (Barua et al., 2001; Pasini et al., 2012). Since microvessels of the blood–brain barrier contain robust antioxidative defence mechanisms, such as superoxide dismutase and glutathione, it is hypothesized that they may provide greater protection against oxidative damage from smoking (Tayarani et al., 1987). Although smoking increases cerebral blood velocity, it has been noted to decrease peripheral artery FMD (Terborg et al., 2002).

Importantly, Terborg et al. (2002) examined the acute effects of smoking, whereas Suzuki et al. (2020) assessed young habitual smokers at rest. Suzuki et al. (2020) utilized a 3‐min steady state cFMD test, and surprisingly, reported no changes in steady state cFMD between young, healthy non‐smokers and habitual smokers. This distinction is important, as the acute cerebrovascular effects of smoking may differ from chronic adaptations observed in habitual smokers under resting conditions. In addition, the relatively short smoking history of the young participants studied by Suzuki et al. (2020) may have limited the degree of chronic vascular impairment present. While smoking consistently impairs systemic endothelial function and reduces peripheral endothelial function, the cerebrovascular circulation may have stronger defences against oxidative stress. However, the work by Suzuki et al. (2020) is the only human study in this area to date, underscoring the need for further research.

## 3 METHODOLOGICAL CONSIDERATIONS

ICA cFMD can be elicited with controlled hypercapnia to increase ICA shear stress and probe endothelial function. This is inherently different from CVR where whole brain haemodynamics can be determined through graded hypercapnic and hypocapnic stages. To determine cerebrovascular endothelial function, two approaches are typically used: transient cFMD tests (brief +6–12 mmHg $P_{\mathrm{ETC}O_{2}}$ steps) that index dynamic reactivity (peak % Δ diameter, time‐to‐peak, SR_AUC_), and steady state cFMD tests (∼3–4 min plateaus at +5 to +10 mmHg). Standardizing these tests is important for providing consistent and translatable measures of cerebrovascular endothelial function. Specifically, both the duration and the magnitude of the CO_2_ stimulus should be considered when investigating cerebrovascular endothelial outcomes.

Extreme levels of $P_{\mathrm{ETC}O_{2}}$ (e.g., >+15 mmHg) could add excessive discomfort, hyperventilation and body movement (which could lead to data variability) during steady state conditions (Willie et al., 2012), but may be more manageable during transient tests, which would reduce variability, though this needs to be confirmed with independent testing. $P_{\mathrm{ETC}O_{2}}$ within a more moderate range (+6, +9, +12 mmHg) yields similar responses once normalized to shear stress during steady state (Sakamoto et al., 2025), but may require specialized respiratory equipment (i.e., end‐tidal forcing) to ensure a robust and rapid enough stimulus is provided during transient tests.

It is worth noting that the onset kinetics of cFMD may be age‐dependent: ICA dilation may be comparable at +5 mmHg across ages, but attenuated in older adults at +10 mmHg (Iwamoto et al., 2018b), with significant differences in the onset of shear stimuli during cFMD tests in children compared with adults (Tallon et al., 2023). More research is required to confirm this age‐related finding, but careful selection of appropriate CO_2_ administration times and recovery imaging periods to ensure that the shear‐mediated response is captured.

Together, these observations argue for moderate CO_2_ steps (∼+7–15 mmHg) during steady state, while greater hypercapnia steps may be feasible during transient tests. Regardless, considering the recent observations by Sakamoto and colleagues about the importance of shear rate stimulus, normalizing the response to blood vessel diameter and shear stress (i.e., SR_AUC_) should be considered when designing cFMD studies (Sakamoto et al., 2025).

Studies utilizing higher CO_2_ stimulus (i.e., >10 mmHg) are cautioned that increases in arterial blood pressure may impact cFMD responses. While pressor responses have been observed in the steady state responses, no transient tests have observed significant hypercapnia‐induced hypertension. Regardless, hypertension can confound findings through hypercapnic hypertensive vascular interactions (Ainslie & Duffin, 2009; Ogoh, 2008). These interactions if unaccounted for and heterogenic during repeated measure testing may elicit unreliable outcomes. Accounting for arterial pressure and utilizing measures of vascular conductance in instances of hypercapnia stimulus eliciting >10 mmHg changes in future cFMD studies is necessary.

## 4 CLINICAL IMPLICATIONS AND FUTURE DIRECTIONS

Although peripheral artery FMD and its prognostic value are well established, the clinical utility of steady state and/or transient cFMD remains uncertain. Robust clinical research – especially longitudinal cohorts and intervention trials – is needed to determine its incremental value for risk stratification and monitoring. To date, most cFMD studies have focused on young, healthy adults, limiting generalizability to higher‐risk groups (e.g., obesity, COPD, diabetes, hypertension). Shorter, transient cFMD protocols may be more feasible than sustained steady state tests, potentially improving tolerability and adherence to hypercapnic stimuli in diseased populations. We therefore propose the following high priority research questions:

1. How should transient and steady state cFMD protocols be standardized (e.g., Δ$P_{\mathrm{ETC}O_{2}}$, stimulus duration, shear stress normalization) to improve reproducibility across laboratories?
2. Is the steady state cFMD response NO‐mediated to the same extent as the transient cFMD response?
3. How do other physiological factors (e.g., core body temperature, neural activity, resting blood pressure) modulate cFMD?
4. Does cFMD predict future cerebrovascular events (e.g., stroke, dementia, cognitive decline) in at‐risk populations?

## 5 CONCLUSION

cFMD in the ICA using hypercapnia to modulate hyperaemia and provoke extracranial cerebrovascular vasodilation has emerged as a promising tool for the assessment of cerebral endothelial function. Evidence to date highlights the sensitivity of ccFMD to exercise, sympathetic activity, oxidative stress, age, sex hormones and lifestyle behaviours, underscoring its value as both a tool to interrogate mechanisms of cerebrovascular function and its potential as a clinical biomarker. However, as outlined in this review, there are still methodological inconsistencies in protocol standardizations, and limited data in ‘at‐risk’ populations, as well as interactions with physiological and environmental stressors. While the potential for cFMD to be equally as important as other measures of cerebrovascular function (i.e., autoregulation and neurovascular coupling), cFMD is a relatively new technique. Its ‘newness’ makes it vulnerable to protocol variations that create experimental noise rather than signal. Thus, standardized approaches are needed for the cFMD technique. This review summarizes two specific techniques used to assess cFMD, and highlights the nuanced differences that make the transient test a bit more relevant in its current form for assessing cerebrovascular endothelial function. Future studies should provide important rationalization when deviating from the approaches discussed in this review in order to reduce experimental noise. Ultimately, this should help produce relevant experimental signals that inform about cerebrovascular endothelial function in brain health. With continued advances in this new and exciting area, cFMD could evolve into a clinically meaningful measure of cerebrovascular health and disease risk.

## AUTHOR CONTRIBUTIONS

All authors were responsible for drafting of the manuscript. All authors have read and approved the final version of this manuscript and agree to be accountable for all aspects of the work in ensuring that questions related to the accuracy or integrity of any part of the work are appropriately investigated and resolved. All persons designated as authors qualify for authorship, and all those who qualify for authorship are listed.

## CONFLICT OF INTEREST

The authors declare no conflicts of interest.
